# Cold Stress Response: An Overview in *Chlamydomonas*

**DOI:** 10.3389/fpls.2020.569437

**Published:** 2020-09-03

**Authors:** Elena Ermilova

**Affiliations:** Biological Faculty, Saint-Petersburg State University, Saint-Petersburg, Russia

**Keywords:** *Chlamydomonas reinhardtii*, low temperature, cold stress, acclimation, chlorophyta

## Abstract

Low temperature (or cold) is one of the major environmental factors that limit the growth and development of many plants. Various plant species have evolved complex mechanisms to adjust to decreased temperature. Mesophilic chlorophytes are a widely distributed group of eukaryotic photosynthetic organisms, but there is insufficient information about the key molecular processes of their cold acclimation. The best available model for studying how chlorophytes respond to and cope with variations in temperature is the unicellular green alga *Chlamydomonas reinhardtii*. *Chlamydomonas* has been widely used for decades as a model system for studying the fundamental mechanisms of the plant heat stress response. At present, unraveling novel cold-regulated events in *Chlamydomonas* has attracted increasing research attention. This mini-review summarizes recent progress on low-temperature-dependent processes in the model alga, while information on other photosynthetic organisms (cyanobacteria and land plants) was used to strengthen generalizations or specializations of cold-induced mechanisms in plant evolution. Here, we describe recent advances in our understanding of cold stress response in *Chlamydomonas*, discuss areas of controversy, and highlight potential future directions in cold acclimation research.

## Introduction

Plants encounter various abiotic stresses, and fluctuations in temperature are among the most common. The capacity to integrate temperature cues and respond appropriately is shared among all plant organisms, ranging from single-celled algae to land plants. Low temperature (or cold) is one of the major environmental factors that limit the growth and development of many plant species (Sanghera et al., 2011; da Cruz et al., 2013; Jeon and Kim, 2013; Chen et al., 2014). Higher plants respond to cold treatment through complex processes at different levels, including physiological modifications and changes in cell membrane lipid composition and concentrations of proteins and metabolites (Kazemi-Shahandashti and Maali-Amiri, 2018). Cold-impacted plants induce the expression of a variety of genes that are controlled by abscisic acid-dependent and -independent pathways (Shi and Yang, 2014). Mesophilic green algae are a widely distributed group of eukaryotic photosynthetic organisms, but there is insufficient information about key physiological and molecular processes of their cold acclimation. In fact, the global cellular responses used by chlorophytes and other microorganisms to survive at low temperatures have been extensively investigated in the past few years with a focus on psychrophilic species (Margesin and Miteva, 2011; Cvetkovska et al., 2017; Collins and Margesin, 2019). The psychrophiles are characterized by the combination of several traits and the distantly related representatives often employ universal adaptations to permanently low temperatures, such as cold-adapted proteins, increased membrane fluidity, antifreeze proteins, and cryoprotectants (Siddiqui et al., 2013). At present, novel omics approaches allow the acquisition of information on the important features of mesophilic green algae to thrive in cold environment (Barati et al., 2019).

The best available model for studying how mesophilic green algae respond to and cope with variations in temperature is *Chlamydomonas reinhardtii*. The sequenced genomes, genetic maps, many molecular tools, and a large indexed mutant library (Merchant et al., 2007; Li et al., 2016; Blaby and Blaby-Haas, 2017) have facilitated the collection of information about the molecular mechanisms underlying the responses of this alga to temperature stresses. *Chlamydomonas* has been studied for decades with regard to heat stress response (Schroda et al., 2015; Rütgers et al., 2017); the process of its acclimation to cold is less understood. The ability of *Chlamydomonas* to adapt to low temperature (*i.e.*, cold acclimation) can be induced at or below 7°C (Maikova et al., 2016). Since temperatures below 3°C lead to vegetative cell death after a short period (Valledor et al., 2013), freezing stress responses have not been analyzed in this alga. Physiologically, the cold-induced response of *Chlamydomonas* manifests as an acclimation phase during which cell division completely stops immediately after the temperature downshift (Lapina et al., 2013). During the cold acclimation phase of many bacteria, archaea, and plants, several cold shock proteins are synthesized, which help the cells to adapt to low temperatures (Phadtare, 2004; Barria et al., 2013; Keto-Timonen et al., 2016). In contrast to higher plants, *Chlamydomonas* contains a single cold shock domain protein (CSP), termed NAB1 (Phytozome 13, *C. reinhardtii* v.5.6), that is not essential for low temperature acclimation (Mussgnug et al., 2005; Sawyer et al., 2015). This implies that cold-specific responses in the alga may be mediated by different, as yet uncharacterized, regulatory mechanisms. At present, unraveling novel cold-regulated events and the underlying transcriptional regulation in *Chlamydomonas* has attracted increasing research attention (Zalutskaya et al., 2019; Li et al., 2020).

This mini-review summarizes recent progress on low-temperature-dependent processes in the model mesophilic alga, while information on other photosynthetic organisms (cyanobacteria and land plants) has been used to strengthen generalizations and specializations in plant evolution. Finally, we conclude that exploring plants with different lifestyles could provide new insights into the evolutionary plasticity of cold-induced adaptation mechanisms.

## Effects of Low Temperature on Different Cell Types in *Chlamydomonas* Life Cycle

**Figure 1:**
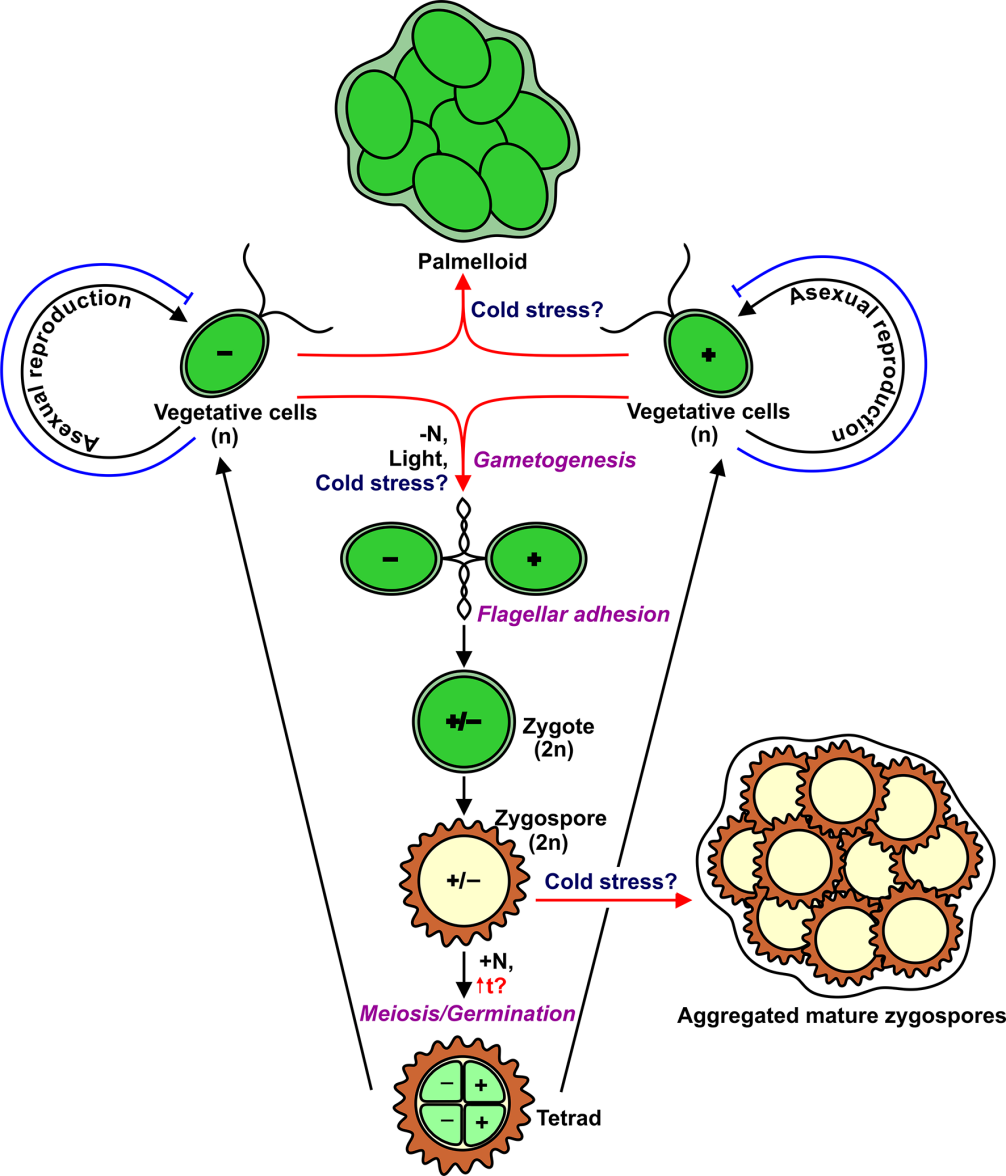
Life cycle of *Chlamydomonas* with indication of multicellular structures such as palmelloids and aggregates. Red arrows show potential cold-induced pathways. Blue T-like lines indicate cold-induced block of cell division.

Within the Chlorophyta, certain genera predominantly form a lichen symbiosis where mycobiont provides suitable conditions for physiological activity and tolerance to stresses of the photosynthetic partner (Karsten and Holzinger, 2012; Míguez et al., 2017). *Chlamydomonas reinhardtii* is a free-living single-celled, biflagellate green alga found in fresh water and soil (Harris, 2009). Vegetative cells are haploid (n) and occur as two mating types, mating type plus (mt^+^) and mating type minus (mt^−^) (**Figure 1**). These cells divide by mitosis when there are adequate environmental conditions (Harris, 2001). The minimal temperature for continued growth of *Chlamydomonas* averages 15–13°C, depending on the media conditions (Maikova et al., 2016). Although cell division completely stops immediately after a temperature downshift (Lapina et al., 2013; Valledor et al., 2013), the cell size continues to increase slowly (Maikova et al., 2016), and the morphology of these cells changes in such a way as a decrease in the density of nucleolus, a change in the chloroplast shape, an increase in the thickness of the starch shell around the pyrenoid, and an increase in the size of vacuoles (Valledor et al., 2013). The expression of a set of genes associated with the cell cycle is downregulated (Li et al., 2020). Notably, three *YAK1*-coding transcripts, the homolog of which in yeast negatively controls cell proliferation, accumulate steadily during low-temperature treatment (Li et al., 2020). Therefore, to some extent, *Chlamydomonas* uses molecular mechanisms to cope with temperature from 15 to 0°C, resulting in viable but not dividing cells. Now, the question is whether *Chlamydomonas* uses cold-adapted cells like cysts as an overwintering form in the natural environment, and if so, whether it can form palmelloids or aggregates (de Carpentier et al., 2019) as an additional mechanism that helps vegetative cells adapt to cold conditions. Similarly, *Chlamydomonas nivalis* and *Haematococcus pluvialis* in response to cold stress become cysts (de Carpentier et al., 2019). Interestingly, in some filamentous green algae (*Klebsormidium*), the cold treatment induces the formation of spores, which show a significantly less susceptibility to photodamage compared to vegetative filaments (Míguez et al., 2020).

In addition to asexual reproduction, *Chlamydomonas* can undergo a sexual cycle (**Figure 1**). In laboratory conditions, gametogenesis is induced by nitrogen depletion in the presence of light, and then gametes of opposite mating types can fuse to form diploid (2n) zygotes that can develop into highly resistant dormant zygospores (Beck and Haring, 1996; Harris, 2001). Importantly, zygospores can survive low temperatures, including freezing (Suzuki and Johnson, 2002). It would be logical to hypothesize that the formation of *Chlamydomonas* zygospores is the most suitable strategy for cold adaptation. However, to form a diploid zygote, motile gametes of the opposite mating type should recognize each other through flagellar adhesion (Harris, 2001). Therefore, the flagella are essential players in the sexual cycle. After a temperature downshift, *Chlamydomonas* cells resorb their flagella for 48–96 h (Valledor et al., 2013; Maikova et al., 2016). The most intriguing questions are which real signals trigger gametogenesis in the environment, and which cellular forms (vegetative cells or zygospores) are typical to survive low temperatures.

## Cold-Induced Signaling Molecules and Regulatory Proteins

**Figure 2:**
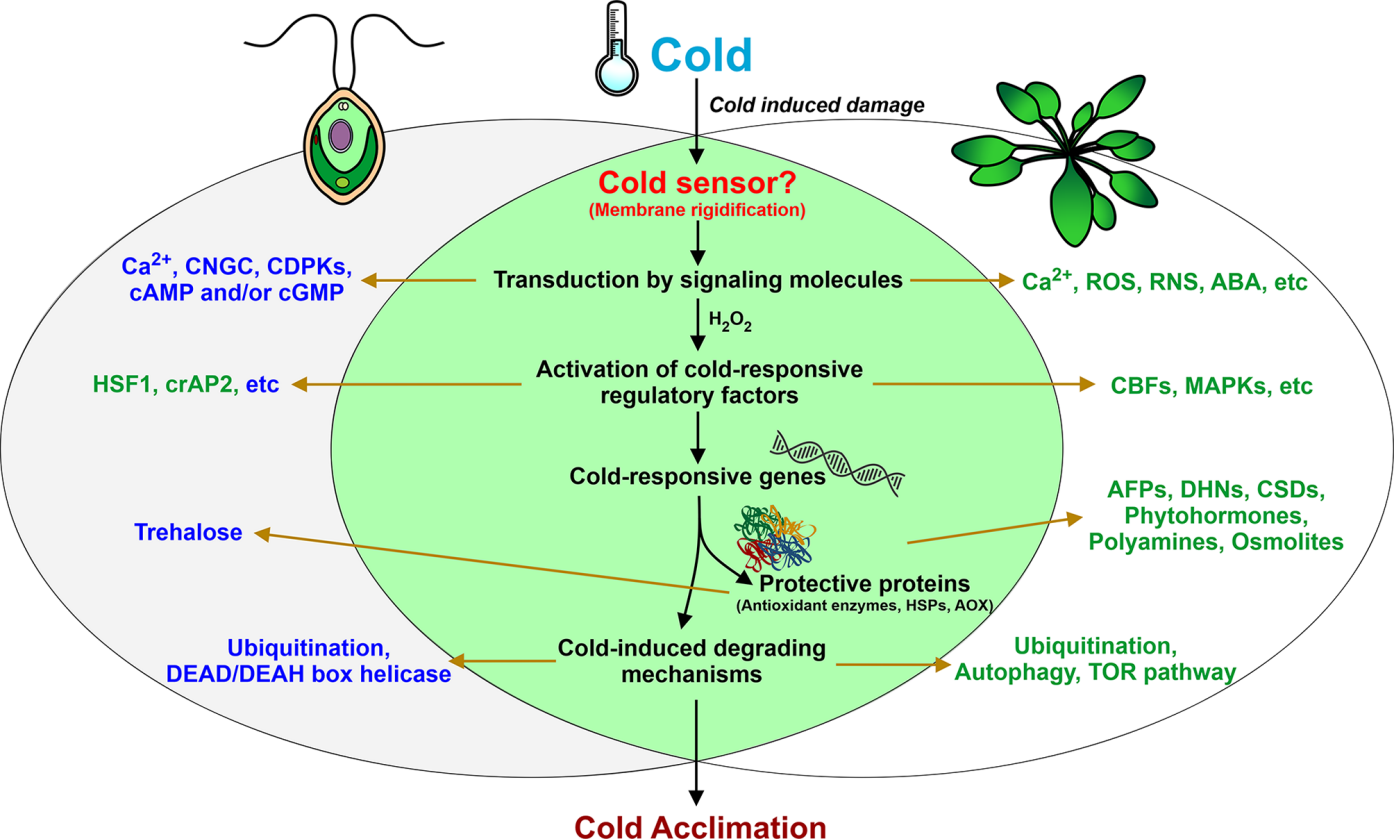
Summary of fundamental responses of *Chlamydomonas* during low-temperature exposure. Left and right circles represent key cold-induced components of *Chlamydomonas* and land plants, respectively. Proven characterized components common to *Chlamydomonas* and land plants are shown in the intersection of two black circles. Components and processes that are potentially involved in *Chlamydomonas* cold acclimation but have been characterized only on the gene expression level are shown in blue. Other proven components are shown in blue.

The key to understanding plant cold response lies in identifying the possible molecular mechanisms of temperature sensing and signaling. Various photosynthetic organisms use different thermosensors to sense temperature changes, including membranes, proteins, nucleic acids, *etc*. (Sinetova and Los, 2016; Kazemi-Shahandashti and Maali-Amiri, 2018). One of the major direct consequences of temperature downshift is a decrease in membrane fluidity affecting membrane-associated cellular functions. In some bacteria, cold sensing occurs *via* changes in the membrane properties by cold sensor histidine kinases, such as DesK of *Bacillus* (Saita and de Mendoza, 2015) and Hik33 of *Synechocystis* (Murata and Los, 2006). In *Chlamydomonas*, to restore membrane flexibility, the amount of polyunsaturated fatty acids is increased (Valledor et al., 2013; Li et al., 2020). Although the exact sensor(s) for the perception of low temperature in this alga is still elusive, the membrane rigidification may be one of the potential mechanisms for activation of the downstream cold-induced signaling pathways (**Figure 2**). Interestingly, this process appears to be one of the first steps in cold stress signaling in *Arabidopsis* (Vaultier et al., 2006).

The next step is transduction of signals into biochemical processes *via* secondary messengers, such as Ca^2+^. In land plants, membrane rigidification can activate mechano-sensitive or ligand-activated Ca^2+^ channels, which leads to an increase in cytosolic Ca^2+^ and subsequent stimulation of the mitogen-activated protein kinase (MAPK) signaling pathway (Knight et al., 1996; Conde et al., 2011). Whether the cytosolic Ca^2+^ increases in response to cold stress in *Clamydomonas* remains to be determined. However, this concept is supported by the finding that transcripts encoding cyclic nucleotide-gated channel (CNGC)-like proteins and calcium-dependent protein kinases (CDPKs) accumulate in *Chlamydomonas* cells after cold treatment (Li et al., 2020). Other secondary messengers, cAMP and/or cGMP, can intertwine with calcium responses to mediate cold-induced signal transduction in the alga (Li et al., 2020).

Cold stress also induces H_2_O_2_ accumulation in plants, including *Chlamydomonas* (O’Kane et al., 1996; Zalutskaya et al., 2019). In various plants, H_2_O_2_ is a key signal molecule that mediates a series of responses (Desikan et al., 2004; Kazemi-Shahandashti and Maali-Amiri, 2018). In *Chlamydomonas*, expression of the cold-responsive gene *GPX3* appears to be regulated by H_2_O_2_ (Zalutskaya et al., 2019). Interestingly, H_2_O_2_ can work in coordination with nitric oxide (NO) in plant cells (Neill et al., 2002; Thakur and Nayyar, 2013**)**. Recent reports indicate that, in diverse plant species, NO plays a crucial role in the control of cold-responsive gene transcription and in post-translational modifications of cold-responsive proteins, namely S-nitrosylation (Puyaubert and Baudouin, 2014). Additionally, in cold-exposed plants, the enhancement of antioxidant defense is achieved *via* different NO-dependent regulatory mechanisms (Farnese et al., 2016). NO is associated with many signaling processes of *Chlamydomonas* (Hemschemeier et al., 2013; Wei et al., 2014; Filina et al., 2019). However, the role of NO in cold signaling in *Chlamydomonas* has not yet been explored.

In cold-stressed plants, there are two main groups of regulatory proteins; those that catalyze dephosphorylation and transcription factors (TFs) (Chinnusamy et al., 2006; Lissarre et al., 2010; Kazemi-Shahandashti and Maali-Amiri, 2018). As mentioned above, *CNGC*- and *CDPK*-coding genes are cold responsive, suggesting the potential involvement of these kinases in cold-induced signaling (Li et al., 2020). Moreover, the plant-specific serine/threonine kinase SnRK2.2, which has a key role in sulfur deprivation responses (Gonzalez-Ballester et al., 2008), is believed to also be involved in the cold acclimation process of *Chlamydomonas* (Unal and Cekic, 2019).

Among the important targets of kinase cascades triggered by cold shock is HSF1 (Maikova et al., 2016), which is a central regulator of the heat stress response in *Chlamydomonas* (Schulz-Raffelt et al., 2007). Another potential TF of regulatory networks in *Chlamydomonas* under low temperature is cold-inducible crAP2 (Li et al., 2020), which belongs to the AP2/EREBP family (Xie et al., 2019). Many TFs from different superfamilies are potential candidates as cold response regulators (Li et al., 2020). However, further studies are required to fully understand which of these represent components of the regulatory networks in cold stress.

## Impacts of Cold on Features Related To Energy Generation

Low-temperature stress affects many aspects of photosynthesis in land plants (Huner et al., 1998; Ensminger et al., 2006; Hajihashemi et al., 2018; Zhu et al., 2018) and green algae (Míguez et al., 2017; Míguez et al., 2020; Zheng et al., 2020). In terrestrial green algae, down-regulation of photochemical processes is a controlled mechanism that favors the dissipation of excess energy to protect the photosynthetic apparatus under low temperature conditions (Míguez et al., 2017). In *Chlamydomonas*, after a temperature decrease, some of the light harvesting complex proteins and b_6_f cytochromes decline, leading to diminished photosynthetic capacity (Valledor et al., 2013). Additionally, although enzymes belonging to the CO_2_ fixation pathway remain at the same level during cold treatment, impaired expression and accumulation of Rubisco and subsequent decreased activity of the Calvin cycle may contribute to overreduction-associated damage to the photosystem and inhibition of the photosynthetic rate (Hashida and Kawai-Yamada, 2019).

In contrast, transcripts of light harvesting chlorophyll a/b stress-related binding protein LHCSR1, which is responsible for nonphotochemical quenching (NPQ) in cells and the PSI-dependent photoprotection mechanism (Kosuge et al., 2018), accumulate during cold exposure (Li et al., 2020). Since NPQ is not decreased in cold-treated *C. reinhardtii* (Zheng et al., 2020), LHCSR1 can also protect the photosynthetic apparatus under low temperatures. Additionally, in cold-treated *Chlamydomonas*, VIPP1, thylakoid formation protein (THF), and monogalactosyldiacylglycerol-specific lipase PGD1 (PLASTID GALACTOGLYCEROLIPID DEGRADATION1) are accumulated (Valledor et al., 2013; Du et al., 2018). VIPP1 and PGD1 appeared to be involved in the organization of thylakoid membranes, where the biogenesis/repair at least of PSII might occur (Rütgers and Schroda, 2013; Du et al., 2018). Interestingly, the enhanced abundance of several photosynthesis-related proteins has been reported under cold stress in *Arabidopsis*, winter wheat, and rice (Janmohammadi et al., 2015). The accumulation of such proteins with the partial recovery of adenosine triphosphate (ATP) synthesis proteins in *Chlamydomonas* suggests the presence of a potential mechanism to reestablish the features of the photosynthetic machinery present in the alga before the onset of cold stress. Further research is needed to validate this hypothesis.

Given that O_2_ solubility increases at low temperatures, increased levels of reactive oxygen species (ROS) are generated, which leads to oxidative stress. Like land plants, *Chlamydomonas* also generates H_2_O_2_ under hypothermia (Zalutskaya et al., 2019). For this reason, as in other unicellular organisms at low temperatures (Tribelli and López, 2018), in this alga, proteins involved in oxidative metabolism such as the tricarboxylic acid cycle (TCA) and the electron transport chain are repressed (Valledor et al., 2013). Notably, in land plants, the TCA is an intermediary and its related enzymes are controlled in an opposite way (Kaplan et al., 2007). On the other hand, glycolysis is maintained at the same level and two key enzymes involved in the pentose–phosphate pathway, 6-phosphogluconolactonase and transaldolase 2, demonstrate increased levels, indicating important features for energy generation in *Chlamydomonas* under cold temperatures (Valledor et al., 2013). No information is available regarding cold-mediated modification in enzymes of glycolysis and the TCA cycle. In spite of this information, additional studies on the consequences of low-temperature stress on glycolytic and respiratory metabolism in Chloroplastida are required.

Many plants degrade their starch reserves to provide energy, sugars, and derived metabolites to mitigate the negative effects of stress (Thalmann and Santelia, 2017). In contrast, in cold-treated *Chlamydomonas* cells, starch is constantly accumulated (Valledor et al., 2013). Furthermore, an increase in starch abundance under stress also occurs in *Arabidopsis* (Kaplan and Guy, 2004; Skirycz et al., 2010) and in its close relative *Thellungiella halophila* (Wang et al., 2013). Although the reason for this difference is unclear, increased starch content is mainly seen in response to high salinity or cold stress (Thalmann and Santelia, 2017). Future research should be directed toward understanding whether it is a species-specific or stress-specific feature.

## Defense Mechanisms and Protective Proteins

Due the imbalanced absorption and utilization of light induced by cold treatment, over-accumulation of ROS occurs in plants (O’Kane et al., 1996; Ensminger et al., 2006; Barati et al., 2019). Some cold-exposed green algae significantly increase the content of photoprotective pigments such as antheraxanthin, zeaxanthin, and total carotenes (Míguez et al., 2020). During cold adaptation, ROS-scavenging enzymes are essential for ROS detoxification (Suzuki and Mittler, 2006; Distelbarth et al., 2013). As in land plants, low-temperature stress induces H_2_O_2_ accumulation in *Chlamydomonas* (Zalutskaya et al., 2019), which may be a result of Rubisco side reactions and Mehler reactions, when sinks for electrons from photosynthetic light reactions become limited (Mehler, 1951).

Notably, among three classes of antioxidant enzymes abolishing the action of H_2_O_2_ [ascorbate peroxidases (APX), catalases (CAT), and glutathione peroxidases (GPX)], *Chlamydomonas* cells under hypothermia induce only *GPX* genes and likely use only one class of antioxidant enzymes for defense of different cell compartments during cold action (Zalutskaya et al., 2019).

Overreduction of the electron transport chain in mitochondria is also a major mechanism of ROS production during temperature stresses (Suzuki and Mittler, 2006). In contrast to animal mitochondria, those of plants possess a branched electron-transport chain with two terminal oxidases, the energy-conserving cytochrome oxidase and the differently termed alternative oxidase (AOX), which is not a proton pump (Affourtit et al., 2002; Finnegan et al., 2004; Millar et al., 2011). Plant AOX prevents overproduction of reactive oxygen species (Vanlerberghe, 2013). In *Chlamydomonas*, AOX1 is a cold-inducible (Molen et al., 2006), suggesting a potential role of this accessory pathway in the hypothermia tolerance of the alga.

In land plants, cold-induced defense mechanisms also include activation of the synthesis of some cryoprotectants such as sugars, proline, glycine betaine, trehalose, and polyamines (Longo et al., 2018). Even though the coordination of biosynthetic gene expression and metabolite accumulation of sucrose and trehalose has been found (Valledor et al., 2013), their protective role in *Chlamydomonas* is questionable, since the increased abundance is small. Additionally, antioxidants such as ascorbic acid and glutathione are not involved in *Chlamydomonas* acclimation to cold stress (Valledor et al., 2013). Moreover, in contrast to other stresses, a shift to low temperature does not induce glycerin, proline, and putrescine synthesis in this alga (Lapina et al., 2013; Valledor et al., 2013). Therefore, whether organic protective molecules are crucial parts of cold adaptation mechanisms in *Chlamydomonas* needs to be investigated.

Another interesting feature of *Chlamydomonas*’s response to temperature downshift is that cold-shock domain nucleic acid binding proteins are not associated with the development of cold tolerance in cells (Mussgnug et al., 2005; Sawyer et al., 2015). These cold-shock proteins function as RNA chaperones (Graumann and Marahiel, 1998; Phadtare, 2004). Whether other proteins exist in algal cells to maintain mRNA in a linear single-stranded form prior to translation remains an open question.

Low temperatures also cause DNA damage in plants (Thakur and Nayyar, 2013). When exposed to cold stress, *Chlamydomonas* may promote cell cycle arrest and DNA repair in order to avoid DNA damage (Li et al., 2020).

In all organisms, the molecular basis of the responses to many different stresses includes rapid accumulation of heat shock proteins (HSPs). Also, in land plants and *Chlamydomonas*, HSPs accumulate in response to low temperatures (Renaut et al., 2006; Timperio et al., 2008; Maikova et al., 2016). Whether other protein families (for example, antifreeze or dehydrin proteins) play a role in the defense mechanisms of *Chlamydomonas* at low temperatures is still unknown (**Figure 2**). Interestingly, *Chlamydomonas* was used to express and secrete a functional antifreeze protein from *Lolium perenne* (*Gramineae*) (Lauersen et al., 2013).

## Cold-Induced Degrading Mechanisms

One of the important consequences of temperature downshift on plants is protein degradation (Kazemi-Shahandashti and Maali-Amiri, 2018). This degradation is essential for the breakdown of misfolded proteins and, as a result, for the production of amino acids and peptides, which can be used to synthesize new proteins that are necessary for adaptation to cold.

Ubiquitination is an important step in nonlysosomal protein degradation in all eukaryotes. Misfolded proteins that are labeled by ubiquitins can then be acted on by proteolytic enzymes called proteasomes. In *Chlamydomonas*, the cold-induced accumulation of RING/U-box superfamily protein (Li et al., 2020) and other cold-specific changes in the ubiquitination system (Valledor et al., 2013) suggest that ubiquitination is involved in cold adaptation. The degradation of abnormal proteins through ubiquitination has also been shown in cold-stressed land plants (Heidarvand and Maali-Amiri, 2013).

Another highly conservative protein degradation system is autophagy (Liu and Bassham, 2012; Michaeli et al., 2016). This catabolic process is mediated by autophagy-related proteins, which have been identified in various organisms, including *Chlamydomonas* (Pérez-Pérez et al., 2010; Pérez-Pérez and Crespo, 2010). In plants, autophagy participates in the responses to many environmental stresses (Wang et al., 2017). Although in *Chlamydomonas*, autophagy is also induced under different stresses (Pérez-Pérez et al., 2017), the potential mechanisms underlying cold-stress-dependent activation of autophagy are still poorly understood (Valledor et al., 2013).

Temperature downshift also leads to changes in secondary structures of RNA and the formation of defective RNA. In *Chlamydomonas*, in the absence of cold-induced CSPs (Mussgnug et al., 2005), RNA helicases and exoribonucleases might stimulate RNA degradation at low temperatures. Indeed, accumulation of the DEAD/DEAH box helicase occurs in algal cells upon cold stress (Valledor et al., 2013). Interestingly, in land plants, members of the DEAD/DEAH box helicase family enhance tolerance to stresses, including cold treatment (Gong et al., 2002; Gong et al., 2005; Vashisht and Tuteja, 2006). Additionally, RNA degradation mediated by exoribonucleases affects plant responses to stresses (Merret et al., 2013). However, unlike bacteria (Phadtare and Severinov, 2010), the role of exoribonucleases in the cold acclimation of plants is still unknown.

## Conclusion and Future Perspectives

*Chlamydomonas* serves as a model plant system to examine many aspects of heat stress response and recently has been used in studies of cold-induced responses; the latter studies have shown that *Chlamydomonas* contains both mechanisms common to other plants and species-specific mechanisms of cold tolerance. Though much work has been done, a lot remains to be done in order to unravel the cold sensor(s) and whole mechanism of signaling and its control. Most of the transcription factors and regulatory proteins for cold-induced signaling network in this alga, often inferred from transcriptomic and metabolomics studies, have not been characterized biochemically. Furthermore, generating and analyzing the molecular genetics of cold-sensitive or cold-tolerant *Chlamydomonas* strains that underexpress or overexpress one or more genes will provide us with an understanding of the basic mechanism of the functioning of stress genes during low-temperature exposure. An outstanding question is related to the relationship between the intensity of the cold (chilling or freezing) and the potential strategies used by this alga in nature (cold-adapted vegetative cells, dormant zygospores, palmelloids, or aggregates). Another interesting avenue will be comparative genomics of psychrophilic *Chlamydomonas* (such as *Chlamydomonas* sp. UWO241, *Chlamydomonas* sp. ICE-L, *Chlamydomonas nivalis*) with that of a representative mesophilic to reveal the unique components or mechanisms present or absent from psychrophiles. We are only starting to have a sense of this, and clearly there is a lot more to learn.

## Author Contributions

The author confirms being the sole contributor of this work and has approved it for publication.

## Funding

This work was supported by a grant from the Russian Science Foundation (grant no.16-14-10004) to EE.

## Conflict of Interest

The author declares that the research was conducted in the absence of any commercial or financial relationships that could be construed as a potential conflict of interest.
